# The Touchstone of Medical Reform; in Three Letters

**Published:** 1841-04-01

**Authors:** 


### MEDICAL REFORM.

I. THE TOUCHSTONE OF MEDICAL REFORM; in Three Letters, addressed to Sir Robert Harry Inglis, Bart. M.P. by *Joseph Henry Green*, F.R.S. Professor of Anatomy to the Royal Academy, one of the Surgeons of St. Thomas's Hospital, &c. Highley, London, 1841.

THIS is not the first time that Mr. Green has appeared before the public as a reformer. This, indeed, is no especial distinction, for all are reformers now-a-days, and the difference lies in the extent to which men go, not in the consent or refusal to move.

Mr. Green's opinions will command attention and deserve much weight. His absence from the field of private practice and of party strife—his independent fortune and position—his high intellectual and eminent professional attainments—and last, not least, the gentlemanly bearing and strict honour of the man, ensure to his sentiments a favourable audience, and remove all suspicion of interest or faction. If a bias can be supposed to stick to opinions emanating from such a source, it can only be the bias of caste, and a leaning towards aristocracy.

We shall content ourselves with putting our readers in possession of the principal views of Mr. Green, without any comments on our part. Events are marching on, and predictions may be falsified before they can be published. Like Lord Melbourne, we feel a particular dislike to venture on prognostications. They are extremely dangerous. Who would have guessed that poor Mr. Hawes's Bill would have vanished so miraculously from the floor of the house? Wise men imitate Lord Burleigh—shake their heads, and say nothing.

Mr. Green's pamphlet consists of Three Letters, intituled respectively—

I. On the Character of a Medical Man in connexion with the Nature and Objects of a Profession.

II. On the Institutions calculated to Educe and Foster the Professional Character.

III. On the Regulation and Economy of the Medical Profession.

I. In setting forth the character of a medical man, Mr. Green lays it down as undeniable that his qualifications should consist in—

1st, The possession of *technical knowledge* and *skill*, in that degree which shall enable each member of the profession to apply all the resources of art, which the whole profession can supply. 2dly, *Scientific insight*, or the possession of the knowledge of those laws or rational grounds, which form at once the principles and ultimate aims of all professional knowledge. And, 3dly, The *character of a gentleman*;—that his conduct shall be the pledge and proof that he pursues his profession as a liberal science, and that, in all his dealings with his patients, his professional brethren, and the community, he is ever guided by the principles of strict professional honour.

Of course this is the ideal of a professional character—what all should aim at, none can attain, but every one will be the better for striving to reach. Mr. Green dwells forcibly on the connexion of medicine with general science, on the necessity of a liberal education, and on the security this offers for the promotion of the philosophy and diminution of the empiricism of physic. And are not the following observations worthy to be read and to be remembered? We think they are.

" It has been my aim to prove the vital connexion between the profession and the science corresponding thereto, so as to establish a balance of sight and insight, between individual skill and the general principles which predetermine its application, and hence likewise the connexion between the profession and the universal sciences, between science itself, and the habit of sciential thought, in the unity of its spirit and essence. It is herein that we find the ground of a *liberal education,* common to the professions, and to the gentry of a country, of an education fitted to maintain the continued succession of a class of *Viri liberales,* of gentlemen, of men imbued with the liberal sciences, of professional men, who in full possession of a liberal science, apply it to the needs and benefits of their fellow citizens. Nor can it be deemed of slight importance, that those destined for the medical profession should partake of that education which is required in common for the liberal professions as an integral part of the gentry of the country, with the sense and habits of a common training in their duties, moral and religious, in their obligations as citizens, and in their sentiments of professional honor as gentlemen. And if the conduct of the medical practitioner is to be the *proof* of his pursuing his profession as the result of a liberal education—of the cultivation of the sciences, as the grounds of the professions, with the common bond in all, that the several sciences are branches of that universal science, the essence of which being the reason tends to give distinct insight, and ultimate aim to all professional knowledges—we may add that the cultivation of science for its own sake, as the predominant object, can alone entitle him to the rank of a gentleman, and must ever constitute the essential difference between a profession and a trade. For, as in the latter, the art is rightfully considered as the exclusive means of gain, so the former must inevitably be degraded into a trade, whenever mercenary and sordid motives supersede the scientific aim. It is not indeed an entire elevation above empirical practice that constitutes the difference between the professional man and the empiric; for both the imperfections and the difficulties of the art, which has so complicated, and at the same time so endlessly variable and fugitive, a subject as the human body in health and disease, will long continue to impose the necessity of practice more or less empirical on the wisest and most profound of the profession. But it is the absence of science, or the contemptuous neglect or disclaiming of the same; it is the elevation of a blind empiricism above science, and as superseding all connexion therewith, that constitutes the empiric, and in all reason degrades him to the carrier on of a trade, a business, or at best an equivocal art. These positions are strictly applicable to the medical profession. We demand of all its members scientific aims and objects; we denounce as empirics those who neglect or disclaim science; we reject as tradesmen those for whom the profession is only a lucrative business; and we brand as quacks those who dishonestly make it the means of levying a tax on the hopes and fears of the ignorant and credulous.

But we say likewise that, as the member of a liberal profession, the medical practitioner is to evince in his whole conduct the character of a gentleman. And it is impossible that the members of the medical profession should have that due weight in society, and occupy that place and rank to which the science entitles them, unless their qualifications and conduct individually, are consonant with the requirements of the professional character, and unless they show by the whole tone and tenor of their conduct and demeanor, that they are fully actuated by its spirit. The character and dignity of the profession, of which each individual member is to be the representative—the education, manners, and habits of those with whom it should be his ambition to associate, namely, those who form the gentry of the country, and constitute its mind by virtue of elevating pursuits, scientific attainments, literary refinement, and moral excellence—and no less the demands of society at large, dictated by that high degree of civilization, to which it has attained in this country, all these challenge those excellencies, which are distinctive of our humanity, and which indeed, are therefore required of every man, but which no calling is more fitted to elicit, and which no calling more imperatively requires, than the medical profession." 16.

II. The Letter on the Institutions calculated to form the Professional Character, is mainly occupied with the same idea, the advantage of connecting the study of medicine with that of general science. The only passage we are inclined to quote, is one referring to the University of London.*

" In alluding to this institution, on the one hand, I should be acting an unworthy part, if I were to flatter the predilections of its unconditional admirers; and on the other hand, I should be unwise to withhold my approbation of an institute, which possesses both the power and the means of exercising the most beneficent control over the future prospects of the medical profession. And I trust, that under its authority, we may yet see the medical schools of the metropolis connected with colleges, in each of which we might find a *school* for elementary instruction, a *senior department* for instruction in those knowledges which are common to all the professions, the proper objects of collegiate education, and heretofore named the liberal arts and sciences, and a *medical department* for the studies properly medical; and these provided with their due appointment of accredited teachers and professors. And I no less fervently hope that the means will be in each case provided of residence within the walls of the college; as I am sure that we cannot estimate too highly the advantages from this provision for an intermediate state between that of the full-grown schoolboy and the independent young man—a state during the most perilous period of human life, in which the individual may remain *sub tutela*, yet no longer as a boy, but as a man influenced by the principles and estimation of his equals, by the example of his seniors, by the habits and laws of the college in which he dwells, and mildly coerced by a peculiar discipline, which even at the time he feels to be an honourable distinction, and which he knows will be hereafter considered by others as entitling him to a distinct rank in society. Lastly, in the co-organization of all the colleges in the unity, and by the bond of universal science, we may hope to find the common grounds of professional excellence gradually reduced to the most effective system, as a complexus of means to various ends, ideally re-united in the same ultimate end." 35.

---

* *Not* University College, but the Metropolitan University.

We admire these aspirations of Mr. Green's. They show his singleness of purpose and integrity of heart. But, so far as the facts have gone, we are not quite sure that anticipations of this sort have been realised. We are not aware that the medical students of King's College or of University College have signalised themselves by their morality or gentlemanly bearing—or that, in these respects, they have proved any better than the pupils of the other schools.

III. The Third Letter comes more to the point than its predecessors, and touches the *raw* of medical reform more sharply.

Mr. Green proclaims himself an enemy to quacks and quackery. Yet he scarcely knows how to go about their demolition.

" It is rather a consideration of the practical difficulties, than any doubt of the principle of interference, that would prevent my urging the Legislature to withdraw its countenance, accorded under a specious and misused plea of liberty, from the knavish quack, and to protect by penal law, his patients, who vindicate their civil rights at the expense of being robbed, maimed, and poisoned. Certainly it is disgraceful to the country that its government should derive a revenue from so unholy a source as that of patents granted to secret remedies and pernicious nostrums. And, at all events, as the writer of the excellent article on Medical Reform in a late number of the Quarterly Review, has pointedly argued, if every individual is to be at liberty to choose his medical adviser in his own case, he ought to be restricted at law from making others the victims of his whims and caprices; and whenevever his functions impose on him the duty of selecting a medical practitioner for a public office, or of appointing him to the charge of others, his choice ought to be limited to those whose qualifications have been tested and approved by the legally constituted authorities." 40.

Mr. Green points out the numerous licensing bodies, their clashing enactments, the want of some controlling power to give harmony to their regulations and direct them to a general rather than a selfish object, and their insufficient powers of protection to their members and repression to impostors. And he adds—

" If then I am right in considering these as the main grounds of the grievances complained of by many members of the profession, and implying a serious drawback on the utility of the medical profession to the community at large, it cannot be donbted that for their rectification and removal, the most pressing need, offered for deliberation to the legislature, is that of forming an efficient *head* for the government of the whole profession, so constituted and vested with such powers as shall secure, under varying circumstances, the unity of the profession, in accordance with its final aim and intention. In considering this important condition, both of the stability of the profession, and of its efficiency, in connexion with the national interests, various plans will suggest themselves, but probably the establishment of a STATE COUNCIL FOR MEDICAL AFFAIRS will be found to be most in harmony with our institutions, and congenial to our habits, and will be best calculated for providing an effective bond of union in regulating and protecting the interests of the different departments of the profession, as *one body*, having an essential community of interests and objects; this council emanating from, and responsible to, the Government of the country for the efficiency of the profession, and for the performance of its duties, private as well as *national*." 44.

" If then the remedy for the evils above adverted to is to be sought in the projected Council of State for medical affairs, the consideration of its constitution and of the mode of its appointment cannot be wholly passed over; and perhaps the following hints may be available in the settlement of the grave questions which these subjects involve, and which must await their final adjustment by the consent of parties seriously affected and variously interested. 1st, As the functions of the Council will be *deliberative,* it should consist, as is indeed implied in the name council, of *various* members; though for the dispatch of business it is evidently desirable that they should be as few as the nature of the constitution of the council permits. 2dly, As representative of the different corporate bodies,—if the principle of representation be adopted in the constitution of the Council, though it is not essential to the plan and involves some serious difficulties—as representative, I say, of the corporations, the proceedings of which are submitted to it for discussion and approval, and as reciprocally influencive of the proceedings of the corporate bodies, which it regulates, the Council might consist of members from each of the medical corporations of the United Kingdom;—whether from all the departments of the profession, whether one or more from each, and whether in like proportions from every one, we leave here undetermined. Thirdly, as the corporate bodies must be best acquainted with the capabilities of their own members and their relative fitness for the duty required, we anticipate no objection to their *nominating* and *recommending* their representative in the Council; but, as the functions of the Council imply pre-eminently *duties* to the profession at large and to the country, we expect that the right of *appointment* will be claimed by the Crown. Fourthly, as the Council is to be the depositary of a trust for the benefit of the nation, and therefore a functionary of the Government, we must anticipate the addition of *Lay Assessors* appointed by the Crown, though the reason assigned does not warrant us in expecting that they will form more than a small proportion of the members, or that they will be other than judicial authorities or legal advisers, and one of the members of Her Majesty's Government." 47.

All *bye-laws* and *ordinances,* emanating from these for the regulation of their own administration, or of the practical departments of the profession, over which they preside, should be submitted to the State Council for its *approval* as the *indispensable condition of their validity.* The accounts of receipt and expenditure of the corporate bodies might be laid before the council.

It may well deserve consideration, whether the duty of taking *cognizance of the practices of unqualified* persons might not be properly committed to it;—and whether, with this view, and as far as this specific object is concerned, it ought not to form a tribunal invested with powers judicial and penal.

Mr. Green hints, too, that all *druggists* and *chemists* and *persons serving* and *compounding medicines,* all *keepers of houses* of reception for *lunatics,* all *dentists, cuppers,* and *the like,* should be obliged to have their qualifications examined, to have a *license* for their several callings, and to be amenable to the State Council for medical affairs. And he conceives that the same tribunal might be armed with authority to expel from the profession all those, who by *dishonourable practices,* had rendered themselves unworthy of the character of members of a liberal profession, whether by the use of secret remedies, by advertizing, by partnerships in trading concerns, by calumnious reports of their professional brethren, by breaches of professional confidence, or by whatever else may be considered derogatory to a professional character.

And this council might attend to the public health, appoint district boards, and arrange medical relief to the poor.

But what is to be done with the present colleges? Are they to be swept away, or preserved and rendered more efficient? Mr. Green argues the matter thus.

"A question meets us at the very threshold of the inquiry, which implicates the safety and continued existence of the metropolitan medical corporations. The main design of these institutions appears to be that of applying to candidates the requisite tests of competency in order to their qualification as practitioners. But it will be said that this has been already done in the cases of Doctors of Medicine at the universities of Oxford and Cambridge; and it may be asked why the graduates of these Universities, with the proof of competency which their degree affords, should be subjected to fresh tests, in order to enable them to practise in London or within seven miles around it. And this question is again the parent of another; why the degree conferred by a University should not be in every instance likewise a license to practise; provided always that its course of instruction be adequate and known, and that it be not suspected of venality in disposing of its degrees. Now this is the main question; for a University has been instituted in London with the main design of conferring medical degrees, and if the foregoing question be answered in the affirmative, it would be difficult to evade the conclusion, or avoid the practical results, that the London University would ultimately become, as has been proposed, the sole source of the licenses to practise, not only of doctors of medicine, but of surgeons, and of general practitioners;—at all events, no very solid reasons could be assigned for the maintenance of the medical corporations of London, if it be true that their functions are confined to ascertaining the qualifications to practise, and that the London University is equally competent to discharge these functions, and is required to perform them in its appointed vocation of conferring degrees. But, specious as this mode of reasoning renders the argument, the truth will be found, I venture to say, in the fact,—implying a very different reply to the question above proposed,—that a university is not competent to perform the duties of the medical corporate bodies. These, indeed, have all the means and appliances of examining the qualifications for a license to practise in their respective departments; and it can scarcely be doubted that in an examination, of which the principal merit is practical, they will have all the advantages derived from the superior attainments of the examiners;—and if a University be better fitted to test the progress of education, preliminary, accessory, and professional, through its different stages, yet it offers no inducements to men of the greatest experience and eminence to contribute their aid in ascertaining its requisite practical sufficiency. It has been, I apprehend, a felicitous result of a London medical education, in connection with its medical corporations, that in giving it throughout a practical tone it has eminently contributed to the character of good sense, which distinguishes the English practice of medicine and surgery. But these corporate bodies, in their nature and design, have other most important functions, conducive to the well-being of the profession over which they preside. They are the *representatives* of the departments of which they are respectively the heads;—they are in their intention the *guardians of the interests* of the profession; and if they have been thought remiss in their duties, it may be fairly attributed rather to a deficiency of their powers than to any want of inclination or zeal;—they are the great organs for promoting the cultivation of the science of the profession, and fitted for collecting and distributing information, the bonds and links of the actual members of the profession throughout England and its vast dependencies, for preserving the unity of the profession in the spirit of ever expanding science, and of professional honor. That their means of exercising these salutary functions might be enlarged cannot be doubted; but we shall in vain look for the same capabilities of maintaining a living intercommunion of the whole actual profession in any institutions, of which the sole purpose terminates in the completion of education." 57.

Mr. Green thinks that it was an error in the authors of a Metropolitan University not availing themselves of the advantages and co-operation of the existing colleges. It is singular that those who hailed the establishment of that university as a death-blow to the colleges, are as loud as ever in their demand for reform and assertion of its necessity—a proof that either such an university is unsatisfactory, or that it has failed in coping with the colleges while the voluntary system operates.

Mr. Green applies himself to the *College of Surgeons* in particular, and specifies the reforms he would see in that.

He is opposed to very popular and open elections in a scientific body. From such would spring, as from a hot-bed, all the evils of intrigue and faction. Evils foul enough in the political world—but in the scientific—

Most foul, strange, and unnatural.

"But," says Mr. Green, "it may be perfectly true that members of the same class as those eligible into the Council may now, from changes in the profession and improvements in education, have become a body, whose attainments and qualifications it would be unwise to overlook or neglect, in any probable change of the constitution of the profession ; and I hold it to be a legitimate object of inquiry whether there are any means of producing (consistently with the design of our institution and the welfare of the profession) a greater confidence in the College, a closer union of its members, and thereby a probable extension of its influence and benefits.

And though there may be no valid reasons for expecting that the functions of the College of Surgeons would be exercised with more advantage to the public or the profession, yet for the purpose of promoting a cordial sympathy and communion of men engaged in common professional objects and having common interests at stake, I would not withhold my assent in any revision of the charter to a modification of the mode of electing members of Council, and to the concession of an *elective privilege,* the conditions of the extension of which beyond the Council I proceed to discuss. And in entering upon the question of these conditions, we dare not for a moment lose sight of the principle, which is to guide us throughout, that the College is essentially a College of Surgeons and unalterably and eminently an institution for the promotion of the *Science of Surgery*. It follows, therefore, undeniably that the first condition of qualification for enjoying the elective franchise is that of *practising Surgery exclusively* ; and, negatively, that the elector neither practises pharmacy nor midwifery, nor belongs to any other college or body incorporated for the promotion of physic, pharmacy or midwifery. That this is not an invidious distinction will be at once apparent, if we consider that the members of the College, who are *Surgeon-apothecaries* form an overwhelming majority, and that making Surgery a subsidiary qualification of their calling, they cannot be supposed to have that interest in the objects, for which the College of Surgeons is instituted, and which the elective trust imperatively requires.

When we consider the mixed character of the members of the College, it is impossible not to see that a broad distinction must ever exist between those who are *Surgeons* by *profession,* and those who make *Surgery a subsidiary qualification :* and in the changes here contemplated we cannot, therefore, doubt the propriety of dividing the two classes, and of distinguishing them by the respective designations of *Fellows* and *Licentiates.* In connexion however with this division, we propose to make it the means and occasion of raising *the standard* of *surgical education* by requiring of the proposed *Fellows* such attainments as shall eminently fit them, not only to be electors but eligible to all places of honor and trust in an institution the object of which, I repeat, is that of promoting the

*science* of surgery :—and in order to prevent any misconception it may be here stated that with exceptions, hereafter adverted to, the qualification for the elective franchise is intended to be twofold, namely, 1st, the practising surgery exclusively,—2nd, the degree and title of *Fellow*."* 62.

The candidate for the fellowship—1st. Should have attained at least twenty-four years of age, though the great and undeniable advantage of a lengthened education are such that the age of twenty-six years would be doubtless a preferable qualification :—2nd. Should have graduated *in Arts* at one of the British Universities, or should show by an examination for that purpose a due proficiency in those branches of study, which that graduation implies, though the Metropolitan Colleges now offer all the requisite facilities for providing the qualification proposed:—3rd. Should be provided with sufficient testimonials of his *moral character* and conduct:—4th. Should be subjected to *examinations* for ascertaining his professional qualifications, which might consist, 1st, Of his acquaintance with the writings of those authors, who mark the great epochs of the *history of* medicine; 2, In *Anatomy* and in *Physiology*, human and comparative;—3, *Pathology*;—4. *Therapeutics*, especially surgical, or what is commonly called the practice of surgery. Moreover he should be required to have occupied six years in his professional education, during a considerable portion of which period he should have attended a hospital, and have treated a certain number of surgical cases;—and he should be required to furnish a series of *clinical reports*.

A Fellow may practise pharmacy or midwifery, but then he could not be an elector of the council. General practitioners who had passed the higher examinations might constitute a class of *Honorary Fellows*, who, showing that they no longer practised pharmacy or midwifery, might claim the privilege of their fellowship.

The *College of Physicians* would be more efficient (Mr. G. does not touch upon its constitution) if its jurisdiction over the practitioners of physic embraced England and its dependencies, in licensing those duly qualified, and in protecting the community from the intrusion of uneducated and dishonest pretenders to medical skill and knowledge.

*A Midwifery Board.*—There would be no difficulty in selecting persons of ability and influence, who might constitute a board for determining the qualifications of those desirous of obtaining a license to practise obstetric medicine, or of those who require it as an additional qualification, and intend to become *Licentiates* in medicine, surgery, and midwifery.

*The Society of Apothecaries.*—" Perhaps the omission of any distinct board of *General Practitioners*, corresponding to the Society of Apothecaries, cannot be wholly passed over without some notice. It is, however, evident that any board

---

* We have heard, we should think the rumour childish, that the College of Physicians have objected to the title of Fellow of the College of Surgeons. They might just as well object to Fellow of the Royal Society, or of the Antiquarian Society, or to the respectable body of *Odd Fellows*. The thing is too absurd to be credited.

for examinations, would be wholly unnecessary, as those described above include the requisite means for determining by those best qualified by their education and attainments, the qualifications of candidates; and we repeat that any separate board would be unnecessary, notwithstanding that we cheerfully admit that the amelioration and improvement of the education of students in London has been mainly owing to the regulations of the Society of Apothecaries. But we see likewise a great evil in the establishment of any separate corporation or governing body for this professional class; and we cannot but think that the general practitioner himself must on reflection see the injurious tendency of any institution, which would be likely to alienate him from those bodies, the character of which tend to give him rank and estimation, and the constitution of which ought to provide inducements and facilities, as is the case in the projected class of Honorary Fellows of the College of Surgeons, for the continual ascension of the general practitioner into the higher grades of the profession, wherever his talents and attainments qualify him for it. It must be likewise remembered that if any such board were established, it must consist of those General Practitioners who live in London. Now in respect of the higher departments of the profession, it is abundantly clear that those of the greatest attainments will be found in the great metropolitan mart of fame and fortune; but for that very reason, the pre-occupation of the posts of honor, namely, it is most likely, as indeed is the fact, that in the class of general practitioners those most eminent in practice, and the most sedulous cultivators of their profession as a science will be found elsewhere than in the metropolis. How little too any hope of founding such an institution in London, in accordance with the requirements of a liberal profession, can be entertained,—will be found in the fact that no feasible means have been, or can perhaps be, devised of separating it from the city guild and *trading company of Apothecaries*. If indeed the medical examinations of general practitioners were conducted by the College of Physicians, and some of the most eminent of the class of general practitioners were selected as *assessors*, it might be with the especial duty of conducting the *pharmaceutical* part of the examination, and the change might be hailed as conferring a legitimate distinction on the individuals, and calculated to exert a beneficial influence prospectively on this indispensable class of the profession. We would wish that a rank and character should be secured to the general practitioner, as a member of a liberal profession, which will be cheerfully conceded to many individuals no less eminent in practice than honourably known as sedulous cultivators of science, but which cannot be granted to them as a body, except under the conditions of an enlarged education, and of the entire separation of their pursuits from any admixture with *Trade*." 70.

Such are the opinions of Mr. Green on Medical Reform. We disclaimed any intention of stating our own views, and conclude, therefore, by recommending those of our author to the candid judgment and criticism of our readers.

